# The Effect of Thermal Fluctuation on the Receptor-Mediated Adhesion of a Cell Membrane to an Elastic Substrate

**DOI:** 10.3390/membranes7020024

**Published:** 2017-04-27

**Authors:** Bahador Marzban, Hongyan Yuan

**Affiliations:** Department of Mechanical, Industrial & Systems Engineering, University of Rhode Island, Kingston, RI 02881, USA; marzban@uri.edu

**Keywords:** cell membrane, fluctuation, Brownian dynamics, Fourier transform, Monte Carlo

## Abstract

Mechanics of the bilayer membrane play an important role in many biological and bioengineering problems such as cell–substrate and cell–nanomaterial interactions. In this work, we study the effect of thermal fluctuation and the substrate elasticity on the cell membrane–substrate adhesion. We model the adhesion of a fluctuating membrane on an elastic substrate as a two-step reaction comprised of the out-of-plane membrane fluctuation and the receptor–ligand binding. The equilibrium closed bond ratio as a function of substrate rigidity was computed by developing a coupled Fourier space Brownian dynamics and Monte Carlo method. The simulation results show that there exists a crossover value of the substrate rigidity at which the closed bond ratio is maximal.

## 1. Introduction

Mechanics of the bilayer membrane play an important role in many biological and bioengineering problems such as membrane remodeling during cell mechanoadaptation [1], cell–substrate and cell–nanomaterial interactions [2], and supported bilayer membranes [3,4,5,6]. In this work, we focus on the effect of thermal fluctuation and the substrate elasticity on the cell membrane–substrate adhesion. Cell–substrate adhesion has attracted considerable interest in past decades due to its importance in mechanobiology as well as in biotechnology where living cells interact with a variety of substrates [7,8,9]. Molecular and cellular mechanisms by which cells “sense” and respond to the mechanical properties of the extracellular matrices can, in general, fall into two categories. In the first category, mechanosensing is actively executed through molecular mechanoenzymatics by which mechanical forces is converted into biochemical signals [10]. The other category is through “passive” physical laws that are derived from the kinetics or energetics of the processes in cell–matrix interactions [11]. Due to the complexity of the biological system, it is very likely that principles from both of these two categories contribute to the cell–matrix interactions. When focusing on the cell–substrate adhesion, it is generally considered that different phases can be distinguished [12]. In the early phase (e.g., the first a few minutes of cell–substrate adhesion), cells come in contact with the substrate to form nascent adhesion patch mainly via receptor–ligand binding, and in the later phases, much more complicated processes are involved such as receptor clustering, cytoskeleton remodeling, and focal adhesion formation [13].

While considerable mechanics modeling efforts have been devoted to the later phase (see a review [11] for more details), the present work is mainly focused on the early phase. The early phase, despite being relatively simpler than the later phase, has also attracted great interest. For example, dedicated vesicle adhesion experiments [14,15] where binding molecules are present in the vesicle membrane and on the substrate have been carried out to mimic cell adhesion. In these experiments, out-of-plane membrane fluctuation was used to detect receptor–ligand binding. In particular, it was found [15] that equilibrium contact zones by ligand–receptor binding were formed and suppressing membrane fluctuation leads to increase of adhesion strength. Furthermore, it has been postulated [16] that cells utilize membrane fluctuation to probe surface properties in the first minutes of cell–substrate contact.

Membrane fluctuation was thought to play an essential role in the early stage of cell adhesion, which is also the central theme of the present work. Besides the membrane–substrate adhesion, membrane fluctuation has also been thought to play important roles in many other cellular processes, such as being utilized to avoid non-specific adhesion [17,18], creating transient gap for actin monomer intercalation during cell migration [19], inducing long-range interactions between membrane proteins or domains [20], and affecting protein mobility on cell membranes [21,22]. In this paper, we aim at developing a quantitative simulation model for the adhesion of the fluctuating membrane and to ascertain whether the membrane fluctuation can be “utilize” to probe the substrate rigidity and receptor density.

## 2. The Model

**Figure 1:**
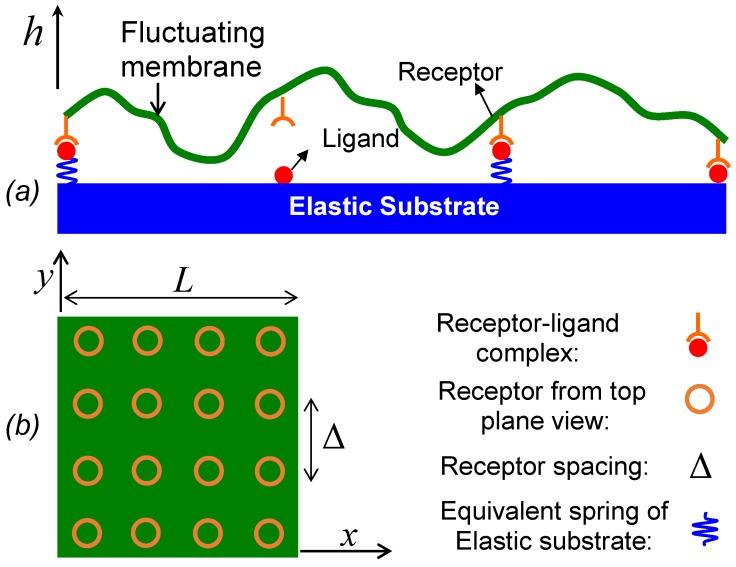
A schematic of a fluctuating membrane adhering to an elastic substrate via ligand–receptor binding: (**a**) side view; (**b**) top view. Note that out-of-plane fluctuation amplitude is exaggerated for better visualization. Structurally, the membrane drawn here includes the glycocalyx layer.

A pioneering work on theoretical modeling of cell adhesion mediated by reversible receptor–ligand bonds was presented by Bell [23], in which the cell adhesion was modeled as a two-step reaction. In the first step, binding proteins diffuse in membranes to form receptor–ligand encounters, and in the second step, receptor–ligand encounters react to form closed bonds. In our case of membrane–substrate, we assume receptors in the membrane are dilute and ligand density on the substrate is saturated, so the effect of the in-plane receptor diffusion on forming receptor–ligand encounters is neglected and receptors are assumed to be immobile and uniformly distributed in the membrane (as illustrated in Figure 1). On the other hand, the cell membrane undergoes out-of-plane undulation as a form of Brownian motion, which brings receptors close to the substrate to form receptor–ligand encounters or inversely pull closed bonds apart. Qualitatively, we can take the same idea as Bell to model the adhesion of a fluctuating membrane as a two-step reaction by simply replacing the in-plane binder diffusion of Bell’s model with the out-of-plane membrane fluctuation,

(1) $$R+L⇄R--L\overset{k_{on}^{0}}{\underset{k_{off}}{⇌}}\;\mathrm{RL}$$

where the first step concerns the formation of encounter complex R--L via membrane fluctuation and the second step is the formation of receptor–ligand bonds with single-molecule reaction rate $k_{on}^{0}$. The forward reaction of the second step can only occur when the ligand–receptor distance is within the encounter distance *R_RL_*. The unbinding rate constant *k_off_* takes Bell’s formula [23] as $k_{off}=k_{off}^{0}\exp\left(fx_{b}/k_{B}T\right)$, where *f* is the force acting on a ligand–receptor bond by the membrane pulling, *x_b_* describes how strongly the reaction rate change with force, $k_{off}^{0}$ is the rate at zero force, *k_B_* is Boltzmann constant, and *T* is the temperature. As showed by Bell [23], for the case of classical particle diffusion, rates of the diffusion step are simple functions of particle diffusivities and encounter distance. In contrast, the two-step reaction in this model is complicated by the membrane fluctuation and we resort to computer simulations. Throughout this paper, we use the closed bond ratio *ϕ*, which is simply defined as the number of closed ligand–receptor bonds divided by the total number of receptors in the membrane, to characterize the adhesion strength. The objective of this paper is to carry out numerical simulations of the chemical reaction in Equation (1) to calculate the closed bond ratio *ϕ*, and to study how it depends on substrate rigidity and receptor density by involving the membrane fluctuation step. It shall be mentioned that previous cell adhesion models [23,24,25,26,27] mainly focused on the rupture strength of a cluster of bonds under an external pulling force, while in our present work, we are concerned with the equilibrium closed bond ratio in the case of no external pulling force.

The fluctuation or Brownian motion of the membrane in potential fields will be simulated using a Fourier space Brownian dynamics (FSBD) scheme developed by Lin and Brown [22,28]. Single ligand–receptor binding-unbinding kinetics will be modeled via Monte Carlo (MC) method and hereafter we refer to this coupled scheme as the FSBD-MC (Fourier space Brownian dynamics- Monte Carlo) simulation. Membrane fluctuation mediated cooperative behavior [29] among different ligand–receptor pairs will be naturally taken into account in the FSBD-MC simulations. On the other hand, particle-based membrane simulation models [30,31,32] are more suitable and straightforward if one needs to simulate receptor diffusion and membrane fluctuation simultaneously. However, in the present work, since receptor diffusion is neglected and only single-valued out-of-plane deformation of a planar membrane is examined, adopting such a Fourier mode-based continuum model can be efficient for the large membranes. The overdamped Langevin equation in Fourier space, with the amplitudes of the undulation modes as the degrees of freedom, reads [28]

(2) $$\frac{\partial h_{q}\left(t\right)}{\partial t}=\Lambda_{q}\left[F_{q}\left(t\right)+\zeta_{q}\left(t\right)\right]$$

where $h_{q}=\int_{A}h\left(x\right)\exp\left(-iq\cdot x\right)dx$ and inversely $h\left(x\right)=L^{-2}\sum_{q}h_{q}\exp\left(iq\cdot x\right)$. As illustrated in Figure 1, x=(x,y), and h(x) is the membrane height, *A* = *L*^2^, *L* is the membrane side length, $\Lambda_{q}=1/\left(4\eta q\right)$, *η* is the viscosity of the surrounding fluid, and q=|q|, q=(2πα/L,2πβ/L), *α* and *β* are integers from 0 to *α*_max_. Note that, in the numerical simulations, two wavelength cutoffs are defined: *λ*_min_ = *L*/*α*_max_ and *λ*_max_ = *L*. The stochastic force $\zeta_{q}\left(t\right)$ has a Gaussian distribution with $\langle \left|\zeta_{q}\left(t\right)\right|\rangle =0$ and $\langle \left|\zeta_{q}\left(t\right)\zeta_{q^{\prime }}\left(t^{\prime }\right)\right|\rangle =2k_{B}TL^{2}\Lambda_{q}^{-1}\delta_{qq^{\prime }}\delta \left(t-t^{\prime }\right)$, and $F_{q}\left(t\right)$ is the deterministic force derived from potential energies which will be described in detail later. Numerical integration is used to update $h_{q}$ in Equation (2) from time *t* to *t* + Δ*t*, and h(x) is obtained by inverse Fourier transform from $h_{q}$. At the end of each time step, $k_{on}^{0}$ is calculated for each open bond if ligand–receptor distance is within the encounter distance *R_RL_*, and $k_{off}=k_{off}^{0}\exp\left(fx_{b}/k_{B}T\right)$ is computed for each closed bond with given bond force *f* calculated based on the membrane position. Then, $k_{on}^{0}\Delta t$ and $k_{off}\Delta t$ are compared with a uniformly distributed random number in [0,1] to determine whether binding or unbinding occurs.

The deterministic force $F_{q}$ in Fourier space is computed from $F_{q}=\int_{A}P\left(x\right)\exp\left(-iq\cdot x\right)dx$, where $P\left(x\right)=-\delta \hat{E}/\delta h$ is the force per unit area acting on the membrane and $\hat{E}$ is the potential or interaction energy per unit area. Next we define all the potential energies involved in the membrane–substrate system illustrated in Figure 1 as follows. First, the membrane bending energy is described by the Canham–Helfrich fluid membrane model [33,34]: $E_{b}$ = $(1/2)\int_{A}B{\left(\nabla^{2}h\right)}^{2}dx$, where *B* denotes bending rigidity, and $\nabla^{2}$ is the Laplace operator in 2D. Thus, the force derived from $E_{b}$ is $F_{q}^{b}=-Bq^{4}h_{q}$. Second, we define the equivalent spring constant *k* of the membrane–substrate linkage (MSL) that takes three components into account: shear deformation resistance in the thickness direction of the lipid bilayer membrane, receptor–ligand complex stiffness, and the substrate elasticity. In this paper, we model the linkage as a linear spring, which is a simplified case for biomaterials that are usually nonlinear. Thus, the potential energy stored in MSL can be written as $E_{L}$ = $\sum_{m=0}^{N_{b}-1}\sum_{n=0}^{N_{b}-1}\int_{A}\frac{1}{2}k{\left(h-h_{L}\right)}^{2}\delta \left(x_{mn}-x\right)\phi_{mn}dx$, where *h_L_* denotes the vertical position of rest unbound ligands, $x_{mn}=\left(m\Delta ,n\Delta \right)$ are uniform locations of ligand–receptor pairs where Δ denotes receptor spacing as shown in Figure 1. The receptor density is then characterized by 1/Δ^2^. The total number of receptors in the domain of interest is denoted by ${\left(N_{b}\right)}^{2}$. The binary function *φ_mn_* describes ligand–receptor binding status with *φ_mn_* = 1 for closed bonds and *φ_mn_* = 0 for open bonds. The force derived from $E_{L}$ is $F_{q}^{L}=-\sum_{m=0}^{N_{b}-1}\sum_{n=0}^{N_{b}-1}k\left[h\left(x_{mn}\right)-h_{L}\right]\exp\left(-iq\cdot x_{mn}\right)$. Since the substrate is a solid bulk material, the thermal fluctuation of the substrate shape is neglected in this model. Therefore, unbound ligands embedded in the substrate are considered to be at rest with the substrate. The elevated ligands elastically restore to their rest positions when unbinding from receptors and such retracting process takes no time by assuming the time scale of elastic restoring is small compared to membrane fluctuation time scale. The substrate is modeled using a soft-wall repulsive interaction $E_{w}$ = $\varepsilon_{w}\int_{A}{\left(\sigma_{w}/\left(h+\sigma_{w}-h_{sub}\right)\right)}^{8}dx$, and *E_w_* is truncated so that $E_{w}\equiv 0$ when *h* > *h_sub_* where *h_sub_* is the upper bound of the repulsive interaction range, *h_sub_* = 0 is assumed in this paper. Here, *σ_w_* and *ε_w_* are parameters determining the repulsive interaction strength. Note that other types of enthalpic repulsive interactions [35], if considered, can also be absorbed into *E_w_*. The force acting on the membrane derived from *E_w_* is

(3) $$F_{q}^{w}=\int_{A}\left[8\left(\varepsilon_{w}/\sigma_{w}\right){\left(\sigma_{w}/\left(h\left(x\right)+\sigma_{w}-h_{sub}\right)\right)}^{9}\right]\exp\left(-iq\cdot x\right)dx$$

## 3. Results

**Figure 2:**
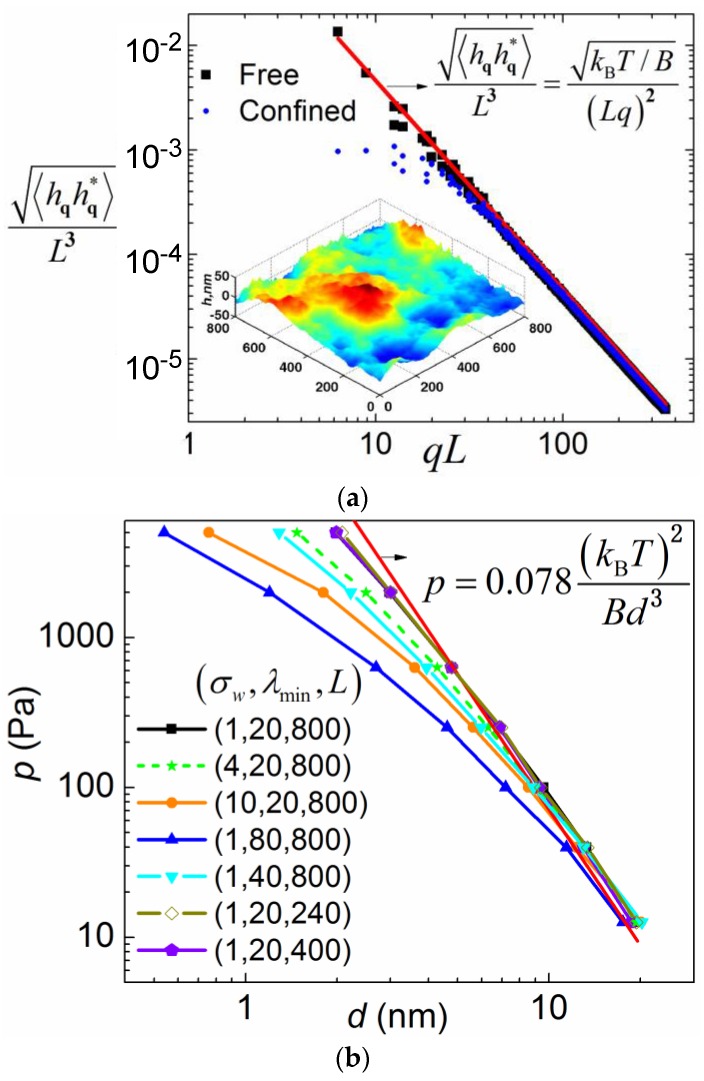
(**a**) Free membrane fluctuation spectrum. Simulation parameters are as follows *L* = 800 nm, *k_B_T* = 4.3 pN·nm, *B* = 20 pN·nm ~ 5 *k_B_T*, *η* = 0.06 Poise, time step Δ*t* = 0.5 ns, total simulation time *t*_total_ = 1 ms, and *λ*_min_ = 20 nm. A simulation snapshot showing fluctuation magnitude and morphology in physics space is plotted as an inset. (**b**) Entropic repulsive interaction between the fluctuating membrane and the substrate. Simulation parameters are as follows: *k_B_T* = 4.3 pN·nm, *B* = 20 pN·nm, *η* = 0.06 Poise, *ε_w_* = 0.043 pN/nm, time step Δ*t* = 0.5 ns, and total simulation time *t*_total_ = 0.6 ms. The triad number denotes $\left(\sigma_{w},\lambda_{\min},L\right)$ in units of nm.

To validate the implementation of the FSBD simulation method, we first carried out free membrane simulations (integrating Equation (2) when $F_{q}\left(t\right)$ = $F_{q}^{b}$), for which a simple analytical expression (the equation inset in Figure 2) exists for the fluctuation spectrum. As shown in Figure 2a, the simulation result (square dots) matches with the theoretical curve (solid line) very well. The equation $\sqrt{<h^{2}(x)>}\approx \sqrt{Ak_{B}T/(4\pi^{3}B)}$ [36] theoretically predicts the mean fluctuation in physics space as ~33 nm for *B* = 20 pN·nm and $\sqrt{A}=L$ = 800 nm, which is comparable to the fluctuation range shown in the inset. The relative mean fluctuation amplitude $\sqrt{<h^{2}(x)>}/L\approx \sqrt{k_{B}T/(4\pi^{3}B)}$ ~4%. The integration time step is chosen so that it is two orders of magnitude smaller than the relaxation time of the deformation mode of the smallest wavelength. For *λ*_min_ = 20 nm, *B* = 20 pN·nm, and *η* = 0.06 Poise, the relaxation time is estimated as [37] $\tau =4\eta \lambda^{3}/B{\left(2\pi \right)}^{3}$~40 ns.

Membrane fluctuation can induce an entropic repulsive force between the membrane and the substrate due to the confinement of the fluctuation [38]. Thus, the membrane is pushed away from the substrate by the entropic pressure. Consequently, the membrane is pinned closer to the substrate on a stiffer substrate by the MSLs for the same amount of closed bonds. We will in the later section show that it is this entropic pressure that gives rise to different closed bond ratios when substrate rigidity is varied. Therefore, it is important to revisit the classical problem of how the entropic pressure is quantitatively dependent on the membrane–substrate distance *d* using the FSBD simulations. Here, instead of considering a fluctuating membrane between two hard walls, we simulate a fluctuating membrane between a soft wall and an externally applied pressure *p* that pushes the membrane against the soft wall. A soft wall is used to make the time–space FSBD numerical simulations applicable. Upon equilibrium, the magnitude of the applied pressure *p* will be equal to that of the entropic pressure. Therefore, we use *p* to represent the entropic pressure in this section. If the membrane is infinitely large and the hard wall is assumed, the only length scale involved is the distance *d*. A simple dimensional analysis yields the following relation between *p* and *d*,

(4) $$p=c\frac{{\left(k_{B}T\right)}^{2}}{Bd^{3}}$$

where *c* is the coefficient to be determined [36,39]. Substantial work has been devoted to determine the coefficient *c* (see ref. [40] and also refs cited therein). Here, we carry out the FSBD simulations by integrating Equation (2) with deterministic forces $F_{q}\left(t\right)$ = $F_{q}^{b}$ + $F_{q}^{w}$+$F_{q}^{p}$, where $F_{q}^{p}=-L^{2}p\delta_{q,0}$ to determine the coefficient *c*. Three more length scales are introduced in our simulations compared to the ideal case: the upper cutoff of the Fourier mode wavelength, which is side length *L*, the lower cutoff wavelength *λ*_min_, and σ*_w_* from the soft wall interaction.

We investigated how the simulation result deviates from Equation (4) when these three length scales are varied. We found that the *p* – *d* relation deviates from Equation (4) drastically when *λ*_min_ is varied. This is rather counterintuitive, because small wavelength modes with relatively small fluctuation seem to be less affected by the hard wall confinement. Varying membrane periodic size from 800 nm to 240 nm only results in negligible changes in the *p* – *d* relation as indicated by the overlapping three curves, as shown in Figure 2b. In addition, increasing the length scale parameter σ*_w_* renders the wall potential softer and thus causes the *p* – *d* relation to deviate from Equation (4). By fitting the simulation data that is closest to Equation (4), we obtain *c* ~ 0.078, which is in good agreement with other simulation results (see the references cited in [40]). Note that, due to the entropic repulsive force, a weak pressure *p*_0_ pushing the membrane towards the substrate is necessary in our FSBD-MC simulations below to prevent the membrane from diffusing far away from the substrate in dynamic processes.

**Figure 3:**
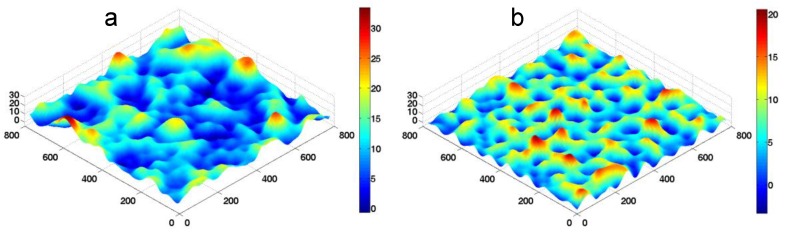
Snapshots of fluctuating membranes in the FSBD-MC simulations. (**a**) *k* = 1 pN/nm; (**b**) *k* = 100 pN/nm.

Next, the FSBD-MC simulations were carried out to ascertain the effect of the substrate rigidity and the receptor density on the membrane adhesion. In this case, $F_{q}\left(t\right)$ = $F_{q}^{L}$ + $F_{q}^{b}$ + $F_{q}^{p}$ + $F_{q}^{w}$ is used in Equation (2). We first briefly summarize the value of the parameters used in the simulations. The second step in Equation (1) in the case of zero bond force is a two-state reaction and the equilibrium constant $k_{on}^{0}/k_{off}^{0}$ is approximated to be $\exp\left(\varepsilon_{b}/k_{B}T\right)$ [41], where *ε_b_* is the ligand–receptor binding energy. Assuming *ε_b_* to be 10 *k_B_T* yields $k_{on}^{0}/k_{off}^{0}$~10^4^. In the literature, $k_{on}^{0}$ was estimated to be from 10^−3^ to 1 ns^−1^ for hapten-antibody [23], and $k_{off}^{0}$ was measured to be from 10^−11^ to 10^−6^ ns^−1^ for integrins [42,43]. Since varying the substrate Young’s modulus is equivalent to changing the MSL spring constant *k* if other segments of the linkage remain unchanged, the lumped spring stiffness *k* is used to represent the change of substrate stiffness. Typical morphologies of the fluctuating membrane in our FSBD-MC simulations are shown in Figure 3.

**Figure 4:**
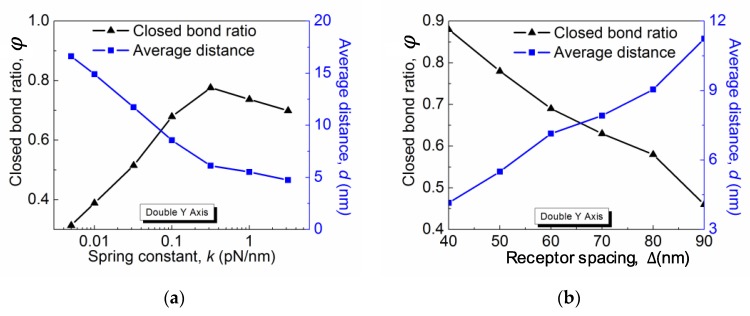
The closed bond ratio as functions of (**a**) the spring constant *k* and (**b**) the receptor density. Simulation parameters are as follows: *p* = 4 × 10^−5^ pN/nm^2^, *h_L_* = −1.2, *σ_w_* = 4 nm, and *ε_w_* = 0.01 *k_B_T*/nm^2^.

The closed bond ratio *ϕ* as a function of the spring constant *k* for different $k_{on}^{0}$ and $k_{off}^{0}$ is plotted in Figure 4a. In all cases, *ϕ* first increases with *k* and then decreases. Such a general behavior can be interpreted as a result of the competition between the change of the rebinding rate and the unbinding rate. Qualitatively, the rebinding rate decreases with the average membrane fluctuation distance, which is characterized here by the average distance *d* between the membrane and the substrate. The distance *d*, as shown on the right *Y*-axis of Figure 4a, decreases with the linkage stiffness *k*, which implies that the membrane is pinned more closely on the stiffer substrate. Therefore, the rebinding rate is larger on a more rigid substrate, which accounts for the increasing region of the *ϕ*-*k* curves. On the other hand, the linkage stiffness *k* also affects the receptor–ligand bond force. Assuming the bond force is induced by the thermal energy, which is constant, the bond force *f* scales with *k* as $f\propto \sqrt{k_{B}T\times k}$. Thus, the bond force increases with *k*, as does the unbinding rate $k_{off}=k_{off}^{0}\exp\left(fx_{b}/k_{B}T\right)$. When the increase in the unbinding rate outperforms the rebinding rate, the equilibrium closed bond ratio decreases, which corresponds to the decreasing region of the *ϕ*-*k* curves. Note that such a substrate rigidity-dependent adhesion scenario only exists in a certain parameter range. As for diffusion-limited reactions, in order for the membrane fluctuation to affect the overall reaction in Equation (1), the rates of the second step need to be much faster than the first step. Therefore, in order to see the substrate rigidity effect on the membrane adhesion, we picked the values of $k_{on}^{0}$ and $k_{off}^{0}$ at their upper bound. As shown in Figure 4a for smaller $k_{on}^{0}$ and $k_{off}^{0}$, the rigidity dependence is weakened and even disappear based on our other simulation data that is not shown here. Simpler than the rigidity dependence, the closed bond ratio *ϕ* monotonically decreases with the receptor distance Δ, as shown in Figure 4b. This is obvious because lowering the receptor density directly reduces the maximum closed bonds per unit area and thus results in a greater membrane–substrate distance subject to the entropic repulsive pressure. Therefore, with other parameters remaining constant, a solely decreasing receptor density leads to a smaller rebinding rate and thus a lower closed bond ratio.

## 4. Conclusions

The simulation results presented in this paper show that when thermally excited membrane fluctuation is taken into account in the specific membrane–substrate adhesion, the adhesion strength characterized by the closed bond ratio becomes dependent on the substrate rigidity and receptor density. The fluctuation induced entropic repulsive force provides the means for the system to “probe” these variations. Such dependence is most evident when the “rate” of the membrane fluctuation step dictated by the viscosity of the surrounding fluid and the membrane bending rigidity is much slower than that of the single receptor–ligand binding and unbinding process. For the classical particle diffusion, the diffusion step only changes the forward and reverse reaction rates but not the overall equilibrium constant. However, it was shown here that the membrane diffusion does affect the overall equilibrium and thus the closed bond ratio. For example, there exists a crossover value of the substrate rigidity at which the closed bond ratio is maximal, and the closed bond ratio monotonically increases with the receptor density. In addition, it is interesting to point out that the membrane fluctuation always works to reduce the closed bond ratio compared to a zero-temperature membrane because, in the absence of membrane fluctuation, the equilibrium constant in Equation (1) K = $k_{on}^{0}/k_{off}^{0}$, which is maximal. Therefore, as shown in our additional simulations that are not presented in this paper, when the membrane fluctuating is suppressed or reduced by, for example, increasing the membrane bending rigidity or increasing the membrane tension [16], the adhesion strength increases.
